# Feasibility and Safety of Overtubes for PEG-Tube Placement in Patients with Head and Neck Cancer

**DOI:** 10.1155/2015/612610

**Published:** 2015-04-21

**Authors:** Crispin O. Musumba, Julia Hsu, Golo Ahlenstiel, Nicholas J. Tutticci, Kavinderjit S. Nanda, David van der Poorten, Eric Y. Lee, Vu Kwan

**Affiliations:** Department of Gastroenterology and Hepatology, Westmead Hospital, Westmead, Sydney, NSW 2145, Australia

## Abstract

*Background*. Percutaneous endoscopic gastrostomy (PEG) placement using the “pull” technique is commonly utilized for providing nutritional support in head and neck cancer (HNC) patients, but it may be complicated by peristomal metastasis in up to 3% of patients. Overtube-assisted PEG placement might reduce this risk. However, this technique has not been systemically studied for this purpose to date. *Methods*. Retrospective analysis of consecutive patients with HNC who underwent overtube-assisted PEG placement at Westmead Hospital, Australia, between June 2011 and December 2013. Data were extracted from patients' endoscopy reports and case notes. We present our technique for PEG insertion and discuss the feasibility and safety of this method. *Results*. In all 53 patients studied, the PEG tubes were successfully placed using 25 cm long flexible overtubes, in 89% prophylactically (before commencing curative chemoradiotherapy), and in 11% reactively (for treatment of tumor related dysphagia or weight loss). During a median follow-up period of 16 months, 3 (5.7%) patients developed peristomal infection and 3 others developed self-limiting peristomal pain. There were no cases of overtube-related adverse events or overt cutaneous metastases observed. *Conclusions*. Overtube-assisted PEG placement in patients with HNC is a feasible, simple, and safe technique and might be effective for preventing cutaneous metastasis.

## 1. Introduction

Percutaneous endoscopic gastrostomy (PEG) tube placement is a well-established technique for providing effective long-term enteral nutrition in patients at risk of malnutrition [1, 2]. In patients with head and neck cancer (HNC), PEG tubes are usually placed either prophylactically in anticipation of nutritional problems leading to weight loss that may arise from chemoradiotherapy- (CRT-) induced toxicity (such as xerostomia, mucositis, nausea, and vomiting) or reactively in symptomatic patients at risk of malnutrition (e.g., due to dysphagia or cachexia). In the US alone, it is estimated that over 200,000 PEG procedures are performed annually [3], 5% in patients with HNC [4] (up to 16.9% of these prophylactically) [5]. With modern improvements in therapy, many patients diagnosed with HNC have an excellent prognosis, particularly those with nasopharyngeal squamous cell carcinomas (SCC) [6, 7], and nutritional management plays a pivotal role in achieving this [8, 9]. Furthermore, the efficacy and safety of novel high precision external beam therapies such as intensity modulated radiation therapy (IMRT) depend on weight-based calculations and minimal anatomical variations due to weight loss during treatment. It is therefore crucial that a patient's weight remains relatively stable throughout treatment to avoid replanning, which involves complex radiation recalculations and dose adjustments [10, 11].

PEG tube placement using the “pull” technique in patients with HNC is associated with an overall complication rate of 20–50%, with a major complication rate of 8–30%, and with a mortality rate of 5% [12–15]. Most of these complications are due to PEG site infections, postulated to be due to transoral PEG tube placement, with resultant translocation of oral and hypopharyngeal bacteria due to overgrowth resulting from varying degrees of upper aerodigestive tract obstruction often present in these patients. Clinically overt abdominal wall tumor metastasis has also been reported to occur in 0.5–3% [4, 16, 17], following prophylactic PEG tube placement in the majority [18, 19], often leading to a dismal prognosis (1 year and overall survival rates of 35.5% and 12.9%, resp.). Worryingly, Ellrichmann et al. recently demonstrated that PEG tube placement using the “pull” technique in patients with oropharyngeal/esophageal tumors resulted in a high rate of microscopic local metastasis (defined as demonstration of tumor cells from brush cytology of the PEG incision site). In this study, the rate immediately after placement and at 6-month follow-up was 22.5% and 9.4%, respectively [18]. The most likely mechanism for tumor seeding is direct translocation of malignant cells from the primary tumor site to the PEG site when the tube is pulled across the tumor during placement [18]. Other postulated mechanisms include hematogenous or lymphatic spread or desquamation and migration of tumor cells to the incision site. Patients at highest risk of tumor seeding are older males with large, advanced stage (stages III and IV) and less well-differentiated pharyngoesophageal SCC [4, 18].

Maetani et al. showed in a prospective randomized trial that “pull” PEG insertion using overtubes in HNC patients significantly reduced peristomal infection compared to no overtube use, by avoiding contact between the PEG tube and oropharyngeal cavity [20]. Subsequently, Couto speculated that this method could also potentially be used to decrease the risk of PEG site metastasis in these patients [21], but this has not yet been systematically studied to date. Some have expressed reservations against adopting this technique, due to the possible complications of overtube use, including esophageal ulceration, perforation, mucosal tears, and bleeding [22, 23]. However, modern improvements in overtube designs incorporating new and safer insertion techniques may overcome most of these problems [24]. The aim of this study is to describe our experience in using a new esophageal overtube system, specifically designed for enhanced protection and safety, for performing PEG tube placement HNC patients.

## 2. Materials and Methods

### 2.1. Study Design and Patients

The study and its design were approved by the Sydney West Area Health Service Human Research Ethics Committee. This is a single-center retrospective study that included data collected in a prospective database from the Endoscopy Unit at Westmead Hospital, a large tertiary referral hospital in Sydney, Australia, from June 2011 to December 2013. In our hospital, all patients newly diagnosed with HNC now undergo prophylactic PEG placement prior to commencing IMRT either alone or in combination with chemotherapy (using cisplatin/carboplatin, with or without 5-fluorouracil). We have been routinely using overtubes for placing PEG tubes in these patients since June 2011, primarily as a way of preventing malignant cutaneous seeding at the PEG site. All patients were first reviewed in the gastroenterology outpatient clinic where the rationale for PEG tube placement as well as risks and benefits of the procedure was explained, and written informed consent was obtained. Exclusion criteria were inability or refusal to give informed consent, obstructing HNC, and high risk of difficult airways management or difficult overtube insertion (such as due to altered head and neck anatomy with reduced mouth opening). After PEG tube placement, patients were reviewed by the nutritional support team and by the oncology team until treatment was completed. The PEG tubes were left in situ for a minimum of 3 months. Cutaneous metastasis was defined as clinically overt metastasis at the PEG site during follow-up after PEG placement.

### 2.2. Technique

**Figure 1:**
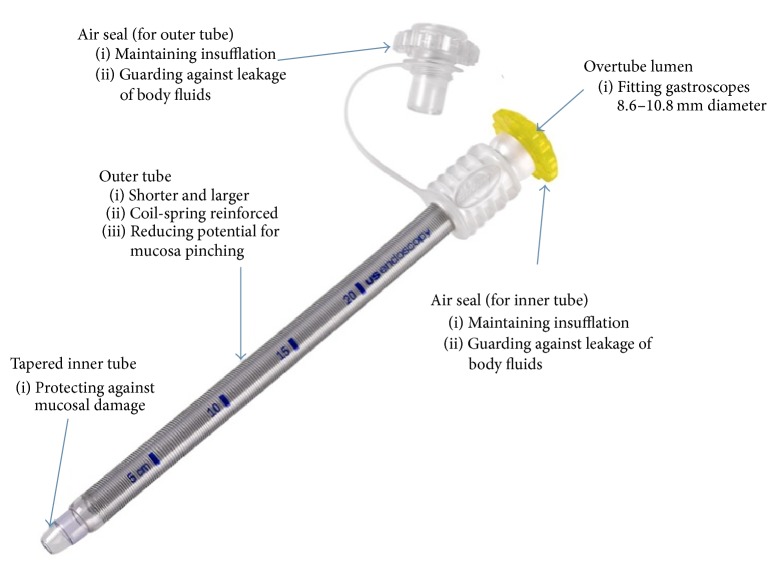
Guardus esophageal overtube (courtesy of US Endoscopy, Mentor, OH).

**Figure 2:**
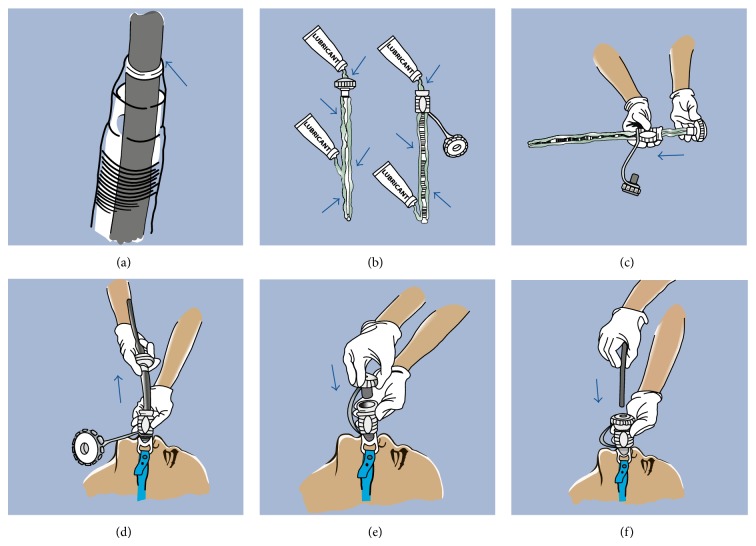
Step-by-step directions for positioning overtube. (a) Guardus overtube with correct snug fit of scope. (b) The inner and outer surfaces of both tubes are generously lubricated using a water-soluble lubricant (not water). (c) The fully lubricated inner tube is inserted into the fully lubricated outer tube and “backloaded” onto the scope, positioning the assembled Guardus overtube at the proximal end of the scope. (d) After performing baseline esophagoscopy, the overtube assembly is gently inserted into the esophagus through the bite block (use of a 60 F bite block is recommended). The inner tube and scope are then simultaneously removed, leaving the outer tube in place. (e) The insufflation cap is attached. This minimizes backflow of bodily fluids and maintains insufflation throughout the procedure. (f) The scope is reintroduced through the insufflation cap into the stomach (courtesy of US Endoscopy, Mentor, OH). PEG placement then proceeds using the standard Guederer-Ponsky “pull” technique, with the catheter pulled through the outer overtube still maintained in the esophagus.

All procedures were undertaken under doctor/nurse-administered conscious sedation using a combination of fentanyl, midazolam, and propofol. When necessary, anesthetic support was utilized in patients deemed to be at high risk of sedation-related complications. Patients were placed in a supine position with the head in a neutral position. All patients received prophylactic antibiotics intravenously using a 3rd generation cephalosporin prior to the procedure. A 25 cm long, single-use, flexible overtube (Guardus; US Endoscopy, Mentor, OH) was used. This overtube system comprises a tapered inner tube that snugly fits around a standard endoscope (8.6–10.8 mm diameter) and an outer slightly shorter and wider coil-spring reinforced tube, both incorporating an air seal at their proximal ends (Figure 1).  Figure 2 demonstrates the step-by-step technique of inserting the Guardus overtube. A standard gastroscope was then introduced with the overtube in place. Subsequent placement of an endoscopically removable 20 F Bard gastrostomy tube (Bard Access Systems, Salt Lake City, UT) was performed using the standard “pull” technique, as previously described by Gauderer and Ponsky [25, 26], with the tube pulled through the overtube [25, 26]. Final position of the PEG tube was confirmed by relook endoscopy, after which the gastroscope and overtube assembly were removed.

### 2.3. Data Acquisition

Data regarding underlying malignancy and indication for PEG tube placement were collated. All endoscopy reports were retrieved from the hospital's endoscopy database (Endoscribe and Provation MD). Details including clinical and demographic parameters, date and indication of PEG tube placement, medications administered during the procedure, description of the procedure, endoscopic findings, and complications were collected. Clinical follow-up data including details of cancer therapy, complications of PEG tube placement, and outcome data were retrieved from case notes and follow-up data records maintained by the nutritional support team and oncology team.

## 3. Results

### 3.1. Patient Demographics

Table 1 summarizes the clinical and demographic characteristics of all the 53 patients who prospectively underwent overtube-assisted PEG placement during the study period. The median age of the patients was 59 years, and 74% were male. PEG tubes were placed prophylactically in 47 (88.7%) patients and reactively in 6 (11.3%) patients (4 with dysphagia, 1 with odynophagia/weight loss, and 1 with tumor related cachexia who was intolerant of a nasogastric tube). 98.1% of the patients received treatment with curative intent comprising chemotherapy and/or radiotherapy in the majority (88.6%), with or without surgery. The most frequent histology of cancer was SCC (96.2%). Most cancers were located in the nasopharynx (28.3%), followed by tongue (20.8%) and tonsils (18.9%). Complete data on tumor staging were available for 27 (50.9%) patients overall; of these 19 (70.4%) had advanced stage disease (stage III or IV). The commonest mode of definitive therapy was chemoradiotherapy (79.2%), followed by surgery (13.2%), either alone or combined with adjuvant chemotherapy.

### 3.2. Procedure Success and Complications

All the 53 patients with HNC included in the study successfully underwent PEG tube placement using the “pull” technique through a Guardus overtube. Procedural results and related complications are shown in Table 2. The mean time to PEG tube removal was 5 months. Postprocedural complications were observed in 7 patients (13.2%), comprising PEG site infection in 3 patients (5.7%), all of which were successfully treated with antibiotics, nonspecific self-limiting peristomal pain in 3 patients (5.7%), and PEG tube dislodgement in 1 patient, leading to premature removal after 5 days. Two patients became PEG-tube dependent, one with poor swallowing after tracheostomy. The median follow-up time after PEG tube placement was 16 months. No patient developed clinically overt peristomal cutaneous metastasis during the follow-up period. There were no procedure-related deaths and all the patients were alive and well at last outpatient follow-up.

## 4. Discussion

In this study we have for the first time demonstrated that PEG tube placement in HNC patients using a flexible overtube and standard “pull” technique is feasible and can be performed safely, with a 100% technical success rate. We did not observe any complications directly related to overtube use, which we attribute to the new overtube design used. The Guardus esophageal overtube system is specifically designed for enhanced protection and safety by protecting against mucosal damage due to pinch injury (Figure 1, http://www.usendoscopy.com/). Hence this system offers a simple, safe, and attractive alternative for safely placing PEG tubes in patients with HNC. Maetani et al. showed that this method significantly reduced the risk of peristomal infection compared to no overtube use and similarly found no overtube-related complications. This is the only study to date that has evaluated overtube-assisted PEG placement [20]. Of note, in their study, only 1 patient (3%) had aerodigestive cancer, and the patients were followed up for only 1 week [20]. In our study, all patients had HNC, the majority with advanced-stage SCC, representing a group of patients at higher risk of major complications (including peristomal infection, cutaneous metastasis). After a median follow-up of 16 months, we observed PEG site infection in 5.7% of the patients, which compares favorably with the rate of 5.4% found in the study by Maetani et al. [20]. There were no cases of PEG site cutaneous metastasis observed, despite the majority of the patients possessing several known high-risk factors for cutaneous metastasis. Since most reported PEG site metastases have been diagnosed after a mean interval of 7.8 ± 5.2 months [4, 19] following PEG placement, we think it is unlikely that any of our patients will develop this complication on longer follow-up. All the patients were still alive at the last time of follow-up.

PEG placement using overtubes in HNC patients using the “pull” technique offers a simple and logical solution for preventing peristomal infection and potentially preventing cutaneous metastasis due to the physical barrier provided by the overtube, which prevents contact of the PEG tube with the oral cavity/hypopharynx bacteria as well as dislodged tumour cells from the primary site. An important advantage of this method is the widespread familiarity of most endoscopists in the use of overtubes. However, this method has not been widely implemented to date primarily due to safety concerns [22]. Previous studies of overtube use in gastrointestinal endoscopy have reported a high rate of complications, including mucosal abrasions and tears and “pinch” injuries of the mucosa between the endoscope and overtube (in up to 72% [27]), esophageal and pharyngeal perforations, variceal rupture, tracheal compression, transient vocal cord paralysis, pneumomediastinum, and overtube separation [23, 24]. However, it is important to note that most of these complications were reported from the use of older overtube designs, which were often larger, stiffer/rigid and had less tapered ends. The newly introduced Guardus esophageal overtube system potentially effectively overcomes most of these complications [24].

The majority (96.6%) of published cases of cutaneous metastasis following PEG placement in patients with HNC to date have occurred following the “pull” technique, supporting a major role played by direct implantation of tumor cells to the PEG incision site in the pathogenesis. It has therefore been proposed that PEG tubes should only be placed in patients with HNC after surgical excision of the primary tumor or, alternatively, by using only those techniques that avoid direct contact between the primary tumor and PEG tube (such as the Russell direct introducer technique [28, 29], radiologically inserted gastrostomy (RIG), or surgical placement). However, there is at present conflicting data on the most ideal method to use. Tucker et al. and Akkersdijk et al. showed a higher rate of both major (including peritonitis, aspiration, PEG site metastasis, necrosis, and abscess) and minor complications (including peristomal infection/pain, tube migration, tube obstruction, and leak) with the “pull” compared to the “push” method in patients with advanced HNC [15, 30]. Conversely, in a recent retrospective analysis, van Dyck et al. showed that using a direct introducer technique with gastropexy compared to a standard pull-through technique in HNC patients resulted in significantly higher complications (48% versus 12%, resp.; *p* < 0.05), including local infection, bleeding, perforation, accidental tube removal, surgery, and mortality [31]. Similarly, use of RIGs in HNC patients has been shown to result in higher morbidity and mortality [32] as well as a higher incidence of tube displacement leading to serious complications [33].

Our study has several limitations. First, in order to avoid contamination, we did not perform endoscopy following final removal of the overtubes after PEG tube placement and hence were unable to assess whether there were any direct overtube-related complications (such as mucosal tears due to pinch injury, ulceration, or bleeding). However, we think if present, these were minor and clinically insignificant/self-limiting, as none of the patients developed overt symptoms. Second, since we excluded patients with advanced stenosing or obstructing HNC, the general applicability of our findings to HNC patients may be limited. However, the majority of HNC patients undergoing PEG placement do so prophylactically, and most do not have advanced obstructing cancers. Moreover, patients with obstructing tumors are potentially at higher risk of overtube-related complications; hence the direct introducer technique should preferentially be used in this group of patients. Third this was a single center, nonrandomized study, involving a small patient cohort, and hence may have been underpowered to detect any cases of overt cutaneous metastasis. Potentially, employing brush cytology of the PEG site immediately following PEG insertion and during follow-up to detect microscopic local metastasis, as has recently been demonstrated [18], could have overcome this problem. Finally, due to its retrospective design, we lacked some data such as the complete tumor stage for some patients.

## 5. Conclusions

In conclusion, the Guardus overtube system provides a simple and safe alternative technique for placing PEG tubes in selected patients with nonobstructing HNC, and it is an attractive option for possibly reducing the risk of peristomal metastasis. Due to the widespread use of overtubes in therapeutic endoscopy and the familiarity most clinicians have in placing PEG tubes using the “pull” method, this technique is well-suited for PEG tube placement in patients with HNC, even in nonspecialized endoscopy units. Further prospective studies in a larger group of patients are needed to confirm our findings and to evaluate the effectiveness of this technique in prevention of cutaneous metastasis.

## Figures and Tables

**Table 1:** Demographic characteristics of 53 patients with head and neck cancer referred to our department for PEG insertion.

	*n* = 53
Median age, years (range)	59 (32–80)
Gender	
Male	39 (73.6%)
Female	14 (26.4%)
Location of HNC	
Nasopharynx	15 (28.3%)
Tongue	11 (20.8%)
Tonsillar	10 (18.9%)
Neck	3 (5.7%)
Palate	3 (5.7%)
Hypopharynx	2 (3.8%)
Vocal cords/glottis	2 (3.8%)
Supraglottic	2 (3.8%)
Cervical lymph nodes	2 (3.8%)
Sinus tract	1 (1.9%)
Olfactory	1 (1.9%)
Larynx	1 (1.9%)
Histology of the HNC	
Squamous cell carcinoma	51 (96.2%)
Neuroendocrine tumor	1 (1.9%)
Neuroblastoma	1 (1.9%)
Stage of tumor^∗^	
Stage II	8 (29.6%)
Stage III	12 (44.4%)
Stage IV	7 (25.9%)
Intent of treatment	
Curative	52 (98.1%)
Palliative	1 (1.9%)
Type of treatment	
Chemoradiotherapy	42 (79.2%)
Surgery + adjuvant chemoradiotherapy	5 (9.4%)
Radiotherapy	3 (5.7%)
Surgery alone	2 (3.8%)
Best supportive care only	1 (1.9%)

HNC: head and neck cancer; PEG: percutaneous endoscopic gastrostomy.

^∗^Complete data on tumor staging was available for 27 (50.9%) patients.

**Table 2:** Procedural results of 53 patients with HNC undergoing successful overtube-assisted PEG placement.

	*n* = 53
Indication for PEG placement	
Prophylactic	47 (88.7%)
Reactive	6 (11.3%)
Mean time to PEG removal, months (range)	5 (3–10)
Median follow-up after PEG placement, months (range)	16 (2–32)
PEG-tube related complications	
Peristomal infection	3 (5.7%)
Peristomal pain	3 (5.7%)
PEG-tube dislodgement	1 (1.9%)
Overtube-related complications	Nil
Overt peristomal cutaneous tumor metastasis	Nil
